# Supplementary material for: A model of the PI cycle reveals the regulating roles of lipid-binding proteins and pitfalls of using mosaic biological data
Source: Sci Rep. 2020 Aug 6;10:13244. doi: 10.1038/s41598-020-70215-7 (PMC7414024; doi:10.1038/s41598-020-70215-7)
Supplement: Supplementary file 1 — Supplementary file1 (PDF 1996 kb) [file 41598_2020_70215_MOESM1_ESM.pdf]

## Supporting Information

# Title:

**A model of the PI cycle reveals the regulating roles of lipid-binding proteins and pitfalls of using mosaic biological data.**

## Authors

Francoise Mazet[1]*, Marcus J. Tindall[2], Jonathan M. Gibbins[1] and Michael J. Fry[1].

## Affiliations

[1] ICMR, School of Biological Sciences, The University of Reading, Whiteknights, Reading RG6 6AS, UK.

[2] Department of Mathematics and Statistics, The University of Reading, Whiteknights, Reading RG6 6AX, UK.

*Correspondence to: f.m.mazet@reading.ac.uk

**This file includes:**

Abbreviations list
Biological rationale and model development
Supplementary Figures S1-S5
Supplementary Tables S1-S7

## Abbreviations list

| | |
|---|---|
| AA | Arachidonic Acid |
| CDIPT | CDP-diacylglycerol--inositol 3-phosphatidyltransferase |
| DAG | Diacylglycerol |
| DAGBP | DAG binding proteins |
| DGK | Diacylglycerol Kinase |
| ER | Endoplasmic Reticulum |
| GPCR | G Protein-Coupled Receptor |
| INPP5 | Inositol-1,4,5-trisphosphate 5-phosphatase |
| Ins | Inositol |
| IP | Inositol phosphates |
| IP2 | inositol 3,4-bisphosphate |
| IP3 | Inositol 1,4,5 trisphosphate |
| IP3Kb | Inositol-trisphosphate 3-kinase B |
| IP3E | IP3 processing enzymes |
| IP4 | inositol tetrakisphosphate |
| LPP | Lipin |
| OCRL1 | Inositol polyphosphate 5-phosphatase 1 |
| OCS | Open Canalicular System |
| P4B | PI4P binding proteins |
| PA | Phosphatidic Acid |
| PABP | PA binding proteins |
| PAP | Phosphatidic Acid Phosphatases |
| PI | Phosphatidylinositol |
| PI4K | Phosphatidylinositol 4-kinase |
| PI4P | Phosphatidylinositol-4-phosphate |
| PI45P2 | Phosphatidylinositol-4,5-bisphosphate |
| PIP5K | Phosphatidylinositol-4-phosphate 5-kinase |
| PL | Phospholipids |
| PLA2 | Cytoplasmic Phospholipase A2 |
| PLB | PI45P2 binding proteins |
| PLC | Phospholipase Cβ |
| R | Gαq-coupled receptor |
| SAC1 | Phosphoinositide phosphatase |
| smG | small G protein |

## Supplementary Text

### Biological rationale and model development.

Plasma membrane PI is in constant flux with phosphatidylinositol 4-phosphate (PI4P) and phosphatidylinositol 4,5-bisphosphate (PI45P2) in the PI cycle. Upon GPCR stimulation, the PLCβ-dependent pathway is immediately activated, hydrolyzing PI45P2 into IP3 and diacylglycerol (DAG). Upon thrombin stimulation, the PLCβ-dependent pathway is immediately activated as indicated by the peaks of inositol 1,4,5-trisphosphate (IP3) and diacylglycerol (DAG) within 5 seconds and is completed within 30 seconds, with the plateauing of both Ins and phosphatidic acid (PA) [7–9] (Supplementary Figure S1, Supplementary Table S1).

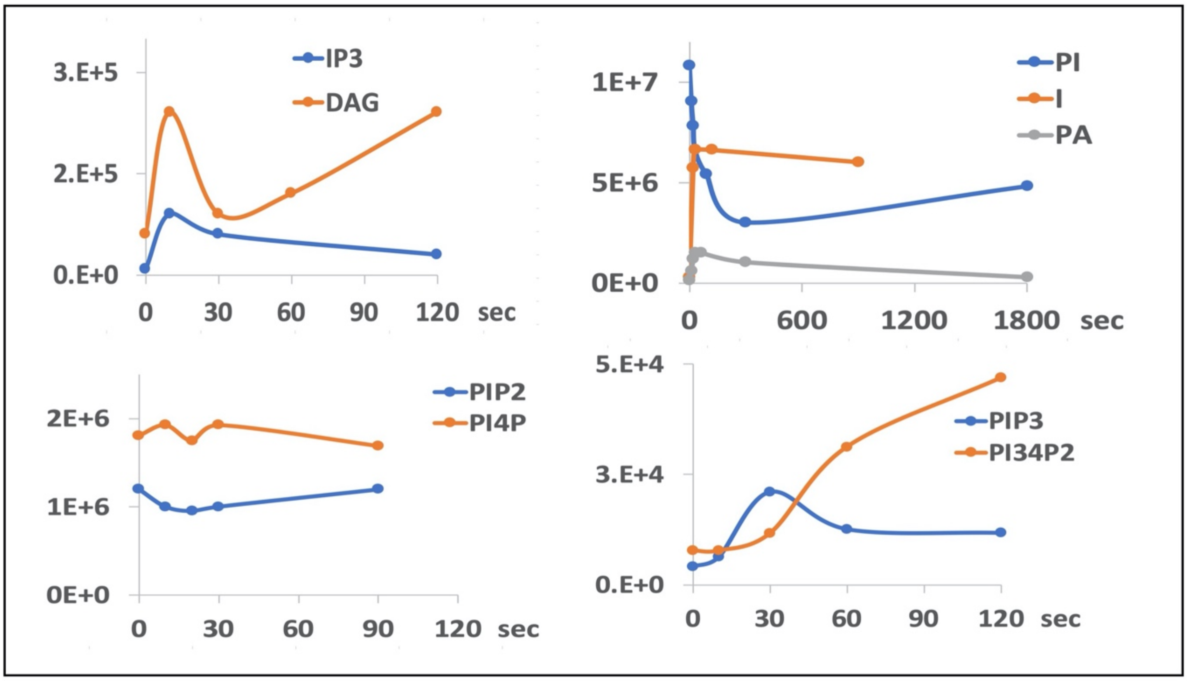


**Supplementary Figure S1. Experimental results**
Collated graphs of experimental data used to inform the model, adapted from (5–10, 22). See Supplementary Table S1 for details and Fig. 2 for standard deviations. Activation occurs at t=0 sec. PIP3 and PI34P2 results are shown for comparison but were not used in our model.

**Table S1:**

| Resting | | | | | | |
|---|---|---|---|---|---|---|
| name | value | unit | molecules/plt | μM* | | References |
| PI45P2 | 1.52 | nmol/10^9 platelets; | 9.15E+05 | 152 | | 16 |
| | 2 | nmol/10^9 platelets | 1.20E+06 | 200 | | 6 |
| PI4P | 3 | nmol/10^9 platelets | 1.81E+06 | 300 | | 6 |
| PI | 18 | nmol/10^9 platelets | 1.08E+07 | 1800 | | 6 |
| | 17.5 | nmol/10^9 platelets | 1.05E+07 | 1750 | | 7 |
| | 62.1 | nmol/5X 10^9 platelets | 7.48E+06 | 1242 | | 8 |
| IP3 | 16 | pmol/10^9 platelets | 9632 | 1.6 | | 16 |
| DAG | 98 | pmol/10^9 platelets | 5.90E+04 | 9.8 | | 11 |
| PA | 1.25 | nmoles 32P/ 5.10^9 platelets | 1.51E+05 | 25 | | 8 |
| PIP3 | 7 | pmol/10^9platelets | 4214 | 0.7 | | 9 |
| PI34P2 | 13 | pmol/10^9platelets | 7826 | 1.3 | | 9 |
| PI3P | 7 | pmol/10^9platelets | 4214 | 0.7 | | 9 |
| PI5P | 15.77 | pmol/mg platelet protein | 3104 | 0.5 | | 14 |
| Inositol | 0.5 | nmol/10^9 platelets | 3.01E+05 | 50 | | 7 |

| Activated | | | | | | |
|---|---|---|---|---|---|---|
| name | value | unit | molecules/plt | μM* | [ligand] | References |
| PI45P2 | 1.5 | nmol/10^9 platelets | 9.03E+05 | 150 | Thrombin 3U/ml | 6 |
| | 148% | % of basal | 1.78E+06 | 296 | Thrombin 1U/ml | 9 |
| | 120% | % of basal | 1.44E+06 | 240 | U46619 5uM | 9 |
| PI4P | 3 | nmol/10^9 platelets | 1.81E+06 | 300 | Thrombin 3U/ml | 6 |
| | 164% | % of basal | *2.96E+06* | *492* | Thrombin 1U/ml | 9 |
| | 129% | % of basal | *2.33E+06* | *387* | U46619 5uM | 9 |
| PI | 8 | nmol/10^9 platelets | 4.82E+06 | 800 | Thrombin 3U/ml | 6 |
| | 7.7 | nmol/10^9 platelets | 4.64E+06 | 770 | Thrombin 10 U/ml | 7 |
| | 35.1 | nmol/5X 10^9 platelets | 4.23E+06 | 702 | Thrombin 0.3 U/10^8 platelets | 8 |
| IP3 | 3.2 | pmol/10^8 platelets | 1.93E+04 | 3.2 | ADP 500 mM | 48 |
| | 134 | pmol/10^9 platelets | 9.03E+04 | 15 | Thrombin 1U/ml | 16 |
| | 200 | pmol/10^9 platelets | 1.20E+05 | 20 | Thrombin 1U/ml | 9 |
| | 170% | % of basal | ND | ND | ADP 20 uM | 25 |
| | 920% | % of basal | ND | ND | Thrombin 1U/ml | 25 |
| DAG | 420 | pmol/10^9 platelets | 2.53E+05 | 42 | Thrombin 1U/ml | 11 |
| PA | 11.11 | nmoles 32P/ 5.10^9 platelets | 1.34E+06 | 222.2 | Thrombin 0.2U/10^8 platelets | 8 |
| | 10.9 | nmol/5X 10^9 platelets | 1.31E+06 | 218 | Thrombin 0.3 U/10^8 platelets | 8 |
| | 216% | % of basal | 3.25E+05 | 54 | ADP 20 uM | 25 |
| | 1730% | % of basal | 2.60E+06 | 432.5 | Thrombin 1U/ml | 25 |
| PIP3 | 300% | % of basal | 1.26E+04 | 2.1 | Thrombin 1U/ml | 9 |
| | 176% | % of basal | 7.42E+03 | 1.232 | U46619 5uM | 9 |
| PI34P2 | 544% | % of basal | 4.26E+04 | 7.1 | Thrombin 1U/ml | 9 |
| | 300% | % of basal | 23478 | 3.9 | U46619 5uM | 9 |
| PI3P | 133% | % of basal | 5.60E+03 | 0.93 | Thrombin 1U/ml | 9 |
| | 123% | % of basal | 5.18E+03 | 0.86 | U46619 5uM | 9 |
| PI5P | 358% | % of basal | 1.11E+04 | 1.8 | Thrombin 1 NIH unit | 14 |
| Inositol | 11 | nmol/10^9 platelets | 6.62E+06 | 1100 | Thrombin 10 U/ml | 7 |

uM* based on a 10 fL platelet

**Supplementary Table S1:** Summary table of published Phospholipids and Inositol Phosphates experimental data.

IP3 is removed very quickly from the cytoplasm by a kinase (IP3Kb) and a PL phosphatase (INPP5). The resulting molecules, inositol tetrakisphosphate (IP4) and inositol 3,4-bisphosphate (IP2) respectively, are then further degraded by a complex set of enzymes (all of which are present in platelets) to produce 6 types of IP before being recycled into Ins [21].

Ins and PA are eventually transported to the ER membrane, where PA is first modified into CDP-DAG and combined with Ins to form a new molecule of PI by the protein complex Nir2-CDIPT-CDS. The resulting PI molecule is then transferred to the plasma membrane by Nir2 in a PI/PA exchange [34]. In this complex, CDIPT is the lowest abundant PI cycle protein in platelets followed closely by Nir2 at 1100 and 1400 copies, respectively. The return of PI, PA and Ins to their initial levels is not observed in thrombin experiments within 15-30 minutes [7], suggesting that the regeneration of PI is rate limiting in these cells. Human platelet temporal changes in PLs and IPs show that around 50% of PI is removed 30 seconds after activation and the amount of Ins produced is roughly equivalent to that amount [6–11] (Supplementary Figure S1). Much of the PI cycle occurs on the plasma membrane while PI is evenly distributed in all cellular membranes [36]. Estimates show that the plasma membrane represents around 54% of the total platelet membrane volume [37]. Hence it is likely that the abrupt cessation of PI conversion by the PLCβ-dependent pathway actually represents the complete depletion of PI in the plasma membrane.

Phosphatidylinositol 4-phosphate (PI4P) and phosphatidylinositol 4,5-bisphosphate (PI45P2) are mostly located in the plasma membrane and are in constant flux with plasma membrane PI in what is known as the "PI futile cycle". PI45P2 and PI4P homeostatic levels are similar at around 1.5 to 1.8 x $10^6$ molecules per platelet while the estimated amount of plasma membrane PI is around 6 x $10^6$ molecules per platelet [6,9]. This suggests unbalanced phosphorylation /dephosphorylation processing between PI and PI4P and balanced processing between PI4P and PI45P2 in inactive cells. Surprisingly, and contrary to PI, their levels vary very little after activation. Recent quantification of PI45P2 in human platelets, however, reveal the presence of a peak of this PL around 30 seconds after Thrombin activation [10]. Similar profiles have been experimentally observed and mathematically

modelled in other cells such as cerebellar Purkinje spines and N1E-115 neuroblastoma cells [2,17,38].

**Model Iteration 1: Inactive PI cycle**

The complexity and diversity of molecules and kinetics in the full signalling pathway, meant obtaining robust estimates of the full model parameter set, given such a large parameter space, would be difficult and unlikely to be well informed given the available data. Using time course data describing the temporal dynamics of PI, Ins, PA and PI45P2 (Supplementary Figure S1, Supplementary Table S1) we started by considering the core reactions involving these lipids, with as many respective reactions removed as possible. This first reduction contained only 4 reactions (Supplementary Figure S2A) that are key to understanding the pathway i.e. the conversion of PI45P2 by PLCβ leading eventually to the production of PA and Ins, the regeneration of PI from PA and Ins by CDIPT and the back-and-forth shuttling between PI and PI45P2 by a simplified set of kinase and phosphatase.

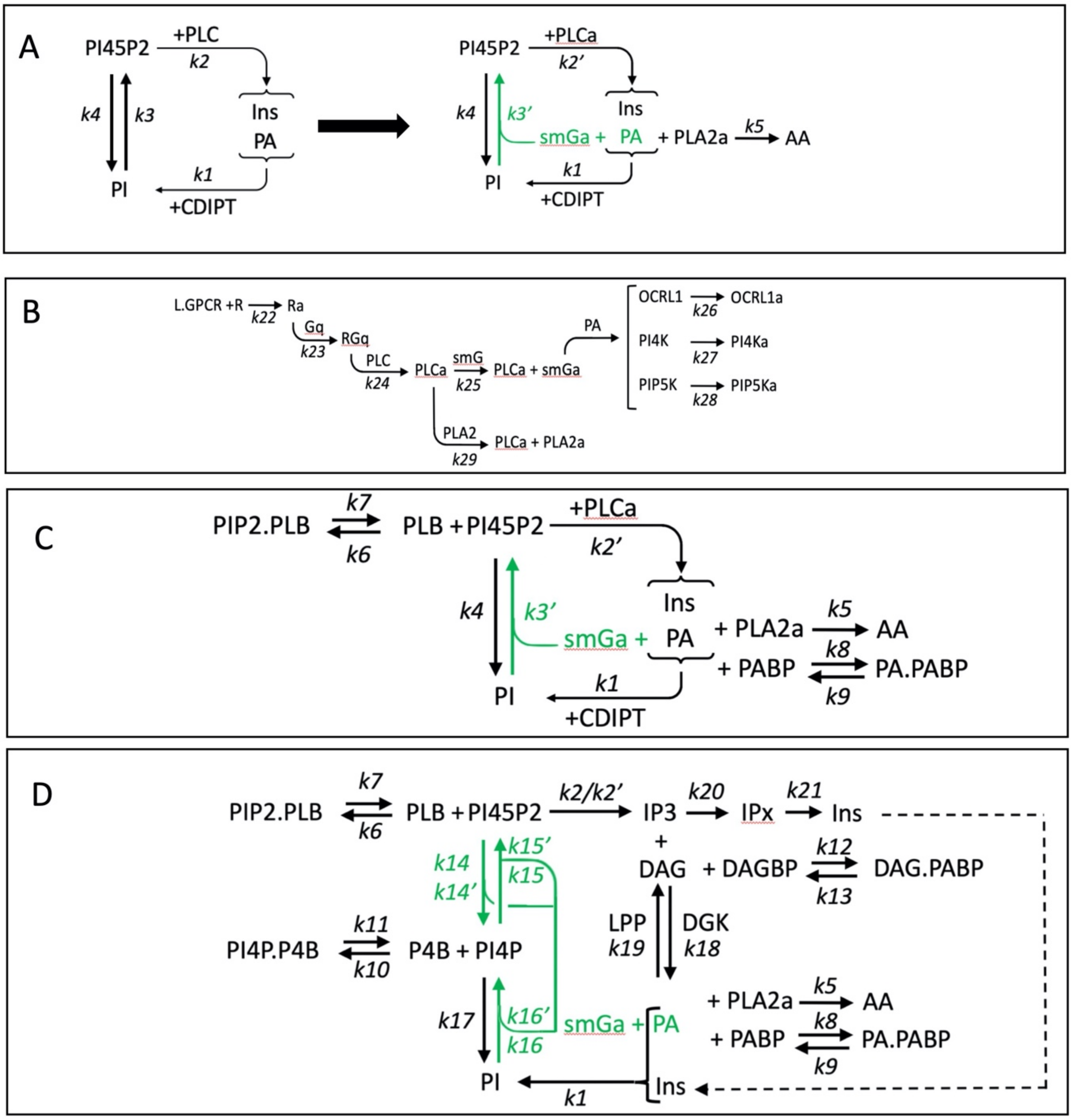


**Supplementary Figure S2. Core model development**

A: Summary diagram of Model Iteration 1 reactions in inactive and activated cells. Only key lipids were kept namely PI, PI45P2, Inositol (Ins) and Phosphatidic Acid (PA). Reactions rates $k_2$ and $k_3$ are replaced by $k_2'$ and $k_3'$ in activated cells, representing the activated rates of the respective enzymes. Coincidence detection leading to the change of rate $k_3$ in $k_3'$ is shown in green. The activation of cPLA2 leads to the removal of some of the PA from the plasma membrane to produce arachidonic acid ($k_5$). $k_1$ and $k_4$ remain unchanged. C: Summary diagram of the addition of lipid binding proteins to regulate the availability of the phospholipids (iteration 3). Although only the activated state is shown in this diagram, the binding and release of the PL was assumed to be constant under both inactivated and activated states of the cell. D: Summary diagram showing iteration 4 of the PI cycle model including the addition of PI4P, DAG and IP3, their respective modifying enzymes and binding proteins. Inactivation reactions not shown. See Supplementary Table S1-S5 for details and parameters.

**Table S2:**

| Uniprot | Name | molecules/plt | Notes | Ref (*= Burkhart, 2012: [5}) | uM |
|---|---|---|---|---|---|
| P62330 | Arf6 | 6400 | | * | 1.06 |
| Q14344 | G13 | 6100 | | * | 1.01 |
| P50148 | Gq | 14800 | | * | 2.46 |
| P23743 | DGKA | 1700 | | * | 0.28 |
| Q16760 | DGKD | | TiO2 fraction | * | |
| P52429 | DGKE | 830 | | * | 0.14 |
| P49619 | DGKG | 2000 | | * | 0.33 |
| Q86XP1 | DGKH | 760 | | * | 0.13 |
| P52824 | DGKQ | 650 | | * | 0.11 |
| Q13574 | DGKZ | 680 | | * | 0.11 |
| Q9BT40 | INPP5K | 800 | | * | 0.13 |
| P27987 | IP3Kb | 1400 | | * | 0.23 |
| Q92539 | LPIN2 | 100 | | * | 0.02 |
| Q01968 | OCRL1 | 850 | | * | 0.14 |
| P51575 | P2X1 | 1400 | | * | 0.23 |
| P47900 | P2Y1 | 134 | | 44 | 0.02 |
| Q9H244 | P2Y12 | 425 | | 45 | 0.07 |
| Q96RI0 | PAR4 | 1100 | | * | 0.18 |
| P25116 | PAR1 | 1276 | 547-2063 | 46 | 0.21 |
| P42356 | PI4KA | 1800 | | * | 0.30 |
| O60331 | PIP5KC | 1200 | | * | 0.20 |
| O14735 | CDIPT | 1100 | | * | 0.18 |
| P47712 | cPLA2 | 3100 | | * | 0.51 |
| Q00722 | PLCB2 | 2500 | | * | 0.42 |
| Q01970 | PLCB3 | 1700 | | * | 0.28 |
| Q15147 | PLCB4 | 1000 | | * | 0.17 |
| P63000 | Rac1 | 32900 | | * | 5.47 |
| P61586 | RhoA | 31300 | | * | 5.20 |
| Q9NTJ5 | SACI | 10500 | | * | 1.74 |
| P21731 | TPa | 1500 | | 47 | 0.25 |

**Supplementary Table S2:** Relevant protein number and UniProt codes.

| Label | Reaction | Reaction Name | Parameters |
|---|---|---|---|
| k1 | PI synthesis reaction | Ins + PA + CDIPT -> PI + CDIPT | k= 7.081 $10^{-4}$ µmol.$s^{-1}$ |
| k2 | PLC reaction resting | PIP2 + PLC -> IP3 + DAG + PLC | k= 0.00025 µmol.$s^{-1}$ |
| k2' | PLC reaction act. | PIP2 + PLCa -> IP3 + DAG + PLCa | k= 0.5 µmol.$s^{-1}$ |
| k5 | cPLA2 reaction | PA + PLA2a -> AA + PLA2a | k= 0.05 $(\mu M.s)^{-1}$ |
| k6/k7 | binding PIP2 | PIP2 + PLB = PIP2.PLB | kf= 0.01325 $(\mu M.s)^{-1}$, kr= 0.75 $s^{-1}$ |
| k8/k9 | binding PA | PA + PABP = PA.PABP | kf= 7.251 $10^{-5}$ $(\mu M.s)^{-1}$, kr= 1.77.$10^{-3}$ $s^{-1}$ |
| k10/k11 | binding PI4P | PI4P + P4BP = PI4P.P4BP | kf= 0.01325 $(\mu M.s)^{-1}$, kr=0.75 $s^{-1}$ |
| k12/k13 | binding DAG | DAG + DAGBP = DAG.BP | kf= 0.01375 $(\mu M.s)^{-1}$ , kr= $10^{-6}$ $s^{-1}$ |
| k14 | OCRL1 reaction rest. | PIP2 + OCRL1 -> PI4P + OCRL1 | k= 0.0001 µmol.$s^{-1}$ |
| k14' | OCRL1 reaction act. | PIP2 + OCRL1a -> PI4P + OCRL1a | k= 3 $(\mu M.s)^{-1}$ |
| k15 | PIP5K reaction rest. | PI4P + PIP5K -> PIP2 + PIP5K | k= 0.001 µmol.$s^{-1}$ |
| k15' | PIP5K reaction act. | PI4P + PIP5Ka -> PIP2 + PIP5Ka | k= 3 $(\mu M.s)^{-1}$ |
| k16 | PI4K reaction rest. | PI + PI4K -> PI4P + PI4K | k= 0.01 µmol.$s^{-1}$ |
| k16' | PI4K reaction act. | PI + PI4Ka -> PI4P + PI4Ka | k= 2 $(\mu M.s)^{-1}$ |
| k17 | SAC1 reaction | PI4P + SAC1 -> PI + SAC1 | k= 0.025 $(\mu M.s)^{-1}$ |
| k18 | DGK reaction | DAG + DGK -> PA + DGK | k= 1 µmol.$s^{-1}$ |
| k19 | LPP reaction | PA + LPP -> DAG + LPP | k= 1 µmol.$s^{-1}$ |
| k20 | IP3 removal | IP3 + IP3E -> IPx + IP3E | k= 9 $(\mu M.s)^{-1}$ |
| k21 | IPx removal | IPx -> Ins | k= 10000 $(\mu M.s)^{-1}$ |
| k22 | activation R | L.GPCR + R -> Ra | k= 1 $(\mu M.s)^{-1}$ |
| k23 | activation Gq | Ra + Gq -> RGq | k= 1 $(\mu M.s)^{-1}$ |
| k24 | activation PLC | PLC + RGq -> PLCa | k= 1 $(\mu M.s)^{-1}$ |
| k25 | activation smG | smG + PLCa = smGa + PLCa | kf= $10^{-5}$ $(\mu M.s)^{-1}$, kr= $10^{-6}$ $s^{-1}$ |
| k26 | activation OCRL1 | OCRL1 + PA + smGa -> OCRL1a | k= 1 µmol.$s^{-1}$ |
| k27 | activation PI4K | PI4K + PA + smGa -> PI4Ka | k= 1 µmol.$s^{-1}$ |
| k28 | activation PIP5K | PIP5K + PA + smGa -> PIP5Ka | k= 1 µmol.$s^{-1}$ |
| k29 | activation PLA2 | PLA2 + PLCa -> PLA2a + PLCa | k= 0.1 µmol.$s^{-1}$ |
| k30 | inactivation Gq | RGq -> RGi | k= 0.02 $(\mu M.s)^{-1}$ |
| k31 | reset PLA2 | PLA2a -> PLA2 | k= 0.01 µmol.$s^{-1}$ |
| k32 | reset OCRL1 | OCRL1a -> OCRL1 + smG + PA | k= 0.003 µmol.$s^{-1}$ |
| k33 | reset PI4K | PI4Ka -> PI4K + smG + PA | k= 0.003 µmol.$s^{-1}$ |
| k34 | reset PIP5K | PIP5Ka -> PIP5K + smG + PA | k= 0.003 µmol.$s^{-1}$ |
| k35 | reset PLC | PLCa -> PLC + RGi | k= 0.003 µmol.$s^{-1}$ |

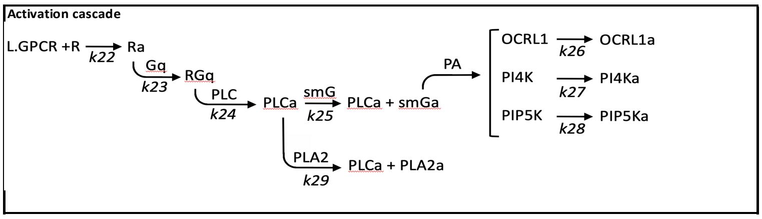


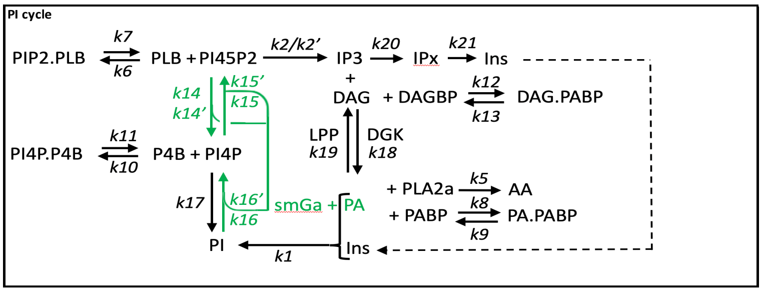


**Supplementary Table S3:** Schematic parameters for the different reactions in iteration 4.

| Name | Particle Number | uM/ initial compartment (= 1fl) |
|---|---|---|
| CDIPT | 1000 | 1.66 |
| DAG | 78.5141 | 0.13 |
| DAG.BP | 36470.3 | 60.56 |
| DAGBP | 25336.7 | 42.07 |
| DGK | 6600 | 10.96 |
| G13 | 6100 | 10.13 |
| Gq | 14800 | 24.58 |
| Ins | 467494 | 776.31 |
| IP3 | 16.4456 | 0.03 |
| IP3E | 3500 | 5.81 |
| IPx | 51.8038 | 0.09 |
| LPP | 100 | 1.66E-01 |
| OCRL1 | 850 | 1.41E+00 |
| OCRL1a | 7.73E-08 | 1.28E-10 |
| P4BP | 143.998 | 2.39E-01 |
| PA | 1.5594 | 2.59E-03 |
| PA.PABP | 161635 | 2.68E+02 |
| PABP | 2.52E+06 | 4.18E+03 |
| PI (pm only) | 6.16E+06 | 1.02E+04 |
| PI4K | 1973.45 | 3.28E+00 |
| PI4Ka | 5.07E-07 | 8.42E-10 |
| PI4P | 461053 | 7.66E+02 |
| PI4P.P4BP | 1.17E+06 | 1.95E+03 |
| PIP2 | 414430 | 6.88E+02 |
| PIP2.PLB | 844497 | 1.40E+03 |
| PIP5K | 1200 | 1.99E+00 |
| PIP5Ka | 1.09E-07 | 1.81E-10 |

| Name | Particle Number | uM/ initial compartment (= 1fl) |
|---|---|---|
| PLA2 | 3000 | 4.98E+00 |
| PLA2a | 0.00139832 | 2.32E-06 |
| PLB | 112 | 1.86E-01 |
| PLC | 5000 | 8.30E+00 |
| PLCa | 1.72E-07 | 2.86E-10 |
| R | 5000 | 8.30E+00 |
| Ra | 3.51E-94 | 5.83E-97 |
| RG13 | 1.06E-27 | 1.76E-30 |
| RGi | 5.61E-09 | 9.32E-12 |
| RGq | 6.60E-73 | 1.10E-75 |
| SAC1 | 10500 | 1.74E+01 |
| smG | 20000 | 3.32E+01 |
| smGa | 5.83E-12 | 9.68E-15 |

**Supplementary Table S4:** Initial particle number and concentrations for human platelets.

**Supplementary Table S5:** Differential Equations

$$\frac{d([PI]\cdot V_{Membrane})}{d t} = +(k1_{("Reaction 01")}\cdot[Ins]\cdot[PA]\cdot[CDIPT]) -(k1_{("Reaction 03")}\cdot[PI]\cdot[PI4K]) +V_{Membrane}\cdot(k1_{("Reaction 06")}\cdot[PI4P]\cdot[SAC1]) -V_{Membrane}\cdot(k1_{("Reaction 03'")}\cdot[PI]\cdot[PI4Ka])$$

$$\frac{d([PI4K]\cdot V_{cytosol})}{d t} = +(k1_{("reset PI4K")}\cdot[PI4Ka]) -(k1_{("activation PI4K")}\cdot[PI4K]\cdot[PA]\cdot[smGa])$$

$$\frac{d([PIP2]\cdot V_{Membrane})}{d t} = -(k1_{("Reaction 05")}\cdot[PIP2]\cdot[OCRL1]) -(k1_{("Reaction 02")}\cdot[PIP2]\cdot[PLC]) -V_{Membrane}\cdot((k1_{("binding PIP2")}\cdot[PIP2]\cdot[PLB]-k2_{("binding PIP2")}\cdot[PIP2.PLB])) -V_{Membrane}\cdot(k1_{("Reaction 05'")}\cdot[PIP2]\cdot[OCRL1a]) +(k1_{("Reaction 04")}\cdot[PI4P]\cdot[PIP5K]) +V_{Membrane}\cdot(k1_{("Reaction 04'")}\cdot[PI4P]\cdot[PIP5Ka]) -(k1_{("Reaction 02'")}\cdot[PIP2]\cdot[PLCa])$$

$$\frac{d([OCRL1]\cdot V_{cytosol})}{d t} = -(k1_{("activation OCRL1")}\cdot[OCRL1]\cdot[PA]\cdot[smGa]) +(k1_{("reset OCRL1")}\cdot[OCRL1a])$$

$$\frac{d([Ins]\cdot V_{cytosol})}{d t} = -(k1_{("Reaction 01")}\cdot[Ins]\cdot[PA]\cdot[CDIPT]) +V_{cytosol}\cdot(k1_{("Reaction 08")}\cdot[IPx])$$

$$\frac{d([PLC]\cdot V_{cytosol})}{d t} = -V_{Membrane}\cdot(k1_{("activation PLC")}\cdot[PLC]\cdot[RGq]) +(k1_{("reset PLC")}\cdot[PLCa])$$

$$\frac{d([PLCa]\cdot V_{Membrane})}{d t} = +V_{Membrane}\cdot(k1_{("activation PLC")}\cdot[PLC]\cdot[RGq]) -(k1_{("reset PLC")}\cdot[PLCa])$$

$$\frac{d([PI4Ka]\cdot V_{Membrane})}{d t} = -(k1_{("reset PI4K")}\cdot[PI4Ka]) +(k1_{("activation PI4K")}\cdot[PI4K]\cdot[PA]\cdot[smGa])$$

$$\frac{d([PIP2.PLB]\cdot V_{Membrane})}{d t} = +V_{Membrane}\cdot((k1_{("binding PIP2")}\cdot[PIP2]\cdot[PLB]-k2_{("binding PIP2")}\cdot[PIP2.PLB]))$$

$$\frac{d([PLB]\cdot V_{Membrane})}{d t} = -V_{Membrane}\cdot((k1_{("binding PIP2")}\cdot[PIP2]\cdot[PLB]-k2_{("binding PIP2")}\cdot[PIP2.PLB]))$$

$$\frac{d([PA]\cdot V_{Membrane})}{d t} = -(k1_{("Reaction 01")}\cdot[Ins]\cdot[PA]\cdot[CDIPT]) +(k1_{("reset PI4K")}\cdot[PI4Ka]) +(k1_{("Reaction 09")}\cdot[DAG]\cdot[DGK]) -(k1_{("Reaction 10")}\cdot[PA]\cdot[LPP]) -(k1_{("activation PIP5K")}\cdot[PIP5K]\cdot[PA]\cdot[smGa]) +(k1_{("reset PIP5K")}\cdot[PIP5Ka]) -(k1_{("activation OCRL1")}\cdot[OCRL1]\cdot[PA]\cdot[smGa]) +(k1_{("reset OCRL1")}\cdot[OCRL1a]) -(k1_{("activation PI4K")}\cdot[PI4K]\cdot[PA]\cdot[smGa]) -V_{Membrane}\cdot(k1_{("Reaction 11")}\cdot[PA]\cdot[PLA2a]) -V_{Membrane}\cdot((k1_{("binding PA")}\cdot[PA]\cdot[PABP]-k2_{("binding PA")}\cdot[PA.PABP]))$$

$$\frac{d([AA]\cdot V_{Membrane})}{dt} = +V_{Membrane}\cdot(k1_{(\text{"Reaction 11"})}\cdot[PA]\cdot[PLA2a])$$

$$\frac{d([PLA2]\cdot V_{cytosol})}{dt} = -(k1_{(\text{"activation PLA2"})}\cdot[PLA2]\cdot[PLCa])$$
$$+(k1_{(\text{"inactivation PLA2"})}\cdot[PLA2a])$$

$$\frac{d([PA.PABP]\cdot V_{Membrane})}{dt} = +V_{Membrane}\cdot((k1_{(\text{"binding PA"})}\cdot[PA]\cdot[PABP]-k2_{(\text{"binding PA"})}\cdot[PA.PABP]))$$

$$\frac{d([PABP]\cdot V_{Membrane})}{dt} = -V_{Membrane}\cdot((k1_{(\text{"binding PA"})}\cdot[PA]\cdot[PABP]-k2_{(\text{"binding PA"})}\cdot[PA.PABP]))$$

$$\frac{d([smGa]\cdot V_{Membrane})}{dt} = -(k1_{(\text{"activation PIP5K"})}\cdot[PIP5K]\cdot[PA]\cdot[smGa])$$
$$-(k1_{(\text{"activation OCRL1"})}\cdot[OCRL1]\cdot[PA]\cdot[smGa])$$
$$-(k1_{(\text{"activation PI4K"})}\cdot[PI4K]\cdot[PA]\cdot[smGa])$$
$$+V_{Membrane}\cdot((k1_{(\text{"activation smG"})}\cdot[smG]\cdot[PLCa]-k2_{(\text{"activation smG"})}\cdot[smGa]\cdot[PLCa])$$

$$\frac{d([smG]\cdot V_{Membrane})}{dt} = +(k1_{(\text{"reset PI4K"})}\cdot[PI4Ka])$$
$$+(k1_{(\text{"reset PIP5K"})}\cdot[PIP5Ka])$$
$$+(k1_{(\text{"reset OCRL1"})}\cdot[OCRL1a])$$
$$-V_{Membrane}\cdot((k1_{(\text{"activation smG"})}\cdot[smG]\cdot[PLCa]-k2_{(\text{"activation smG"})}\cdot[smGa]\cdot[PLCa])$$

$$\frac{d([L.GPCR]\cdot V_{Membrane})}{dt} = -V_{Membrane}\cdot(k1_{(\text{"activation R"})}\cdot[L.GPCR]\cdot[R])$$

$$\frac{d([RGi]\cdot V_{Membrane})}{dt} = +V_{Membrane}\cdot(k1_{(\text{"inactivation Gq"})}\cdot[RGq])$$
$$+(k1_{(\text{"reset PLC"})}\cdot[PLCa])$$
$$+V_{Membrane}\cdot(k1_{(\text{"inactivation G13"})}\cdot[RG13])$$

$$\frac{d([PLA2a]\cdot V_{Membrane})}{dt} = +(k1_{(\text{"activation PLA2"})}\cdot[PLA2]\cdot[PLCa])$$
$$-(k1_{(\text{"inactivation PLA2"})}\cdot[PLA2a])$$

$$\frac{d([DAG]\cdot V_{Membrane})}{dt} = +(k1_{(\text{"Reaction 02"})}\cdot[PIP2]\cdot[PLC])$$
$$-(k1_{(\text{"Reaction 09"})}\cdot[DAG]\cdot[DGK])$$
$$+(k1_{(\text{"Reaction 10"})}\cdot[PA]\cdot[LPP])$$
$$-V_{Membrane}\cdot((k1_{(\text{"binding DAG"})}\cdot[DAG]\cdot[DAGBP]-k2_{(\text{"binding DAG"})}\cdot[DAG.BP]))$$
$$+(k1_{(\text{"Reaction 02'"})}\cdot[PIP2]\cdot[PLCa])$$

$$\frac{d([IP3]\cdot V_{cytosol})}{dt} = +(k1_{(\text{"Reaction 02"})}\cdot[PIP2]\cdot[PLC])$$
$$-V_{Membrane}\cdot(k1_{(\text{"Reaction 07"})}\cdot[IP3]\cdot[IP3E])$$
$$+(k1_{(\text{"Reaction 02'"})}\cdot[PIP2]\cdot[PLCa])$$

$$\frac{d([IPx]\cdot V_{cytosol})}{dt} = +V_{Membrane}\cdot(k1_{(\text{"Reaction 07"})}\cdot[IP3]\cdot[IP3E])$$
$$-V_{cytosol}\cdot(k1_{(\text{"Reaction 08"})}\cdot[IPx])$$

$$\frac{d([OCRL1a]\cdot V_{Membrane})}{dt} = +(k1_{(\text{"activation OCRL1"})}\cdot[OCRL1]\cdot[PA]\cdot[smGa])$$
$$-(k1_{(\text{"reset OCRL1"})}\cdot[OCRL1a])$$

$$\frac{d([PI4P] \cdot V_{Membrane})}{dt} = +(k1_{("Reaction\ 05")} \cdot [PIP2] \cdot [OCRL1])$$

$$+(k1_{("Reaction\ 03")} \cdot [PI] \cdot [PI4K])$$

$$+V_{Membrane} \cdot (k1_{("Reaction\ 05'")} \cdot [PIP2] \cdot [OCRL1a])$$

$$-V_{Membrane} \cdot (k1_{("Reaction\ 06")} \cdot [PI4P] \cdot [SAC1])$$

$$-(k1_{("Reaction\ 04")} \cdot [PI4P] \cdot [PIP5K])$$

$$-V_{Membrane} \cdot (k1_{("Reaction\ 04'")} \cdot [PI4P] \cdot [PIP5Ka])$$

$$-V_{Membrane} \cdot ((k1_{("binding\ PI4P")} \cdot [PI4P] \cdot [P4BP] - k2_{("binding\ PI4P")} \cdot [PI4P.P4BP]))$$

$$+V_{Membrane} \cdot (k1_{("Reaction\ 03'")} \cdot [PI] \cdot [PI4Ka])$$

$$\frac{d([PIP5Ka] \cdot V_{Membrane})}{dt} = +(k1_{("activation\ PIP5K")} \cdot [PIP5K] \cdot [PA] \cdot [smGa])$$

$$-(k1_{("reset\ PIP5K")} \cdot [PIP5Ka])$$

$$\frac{d([PIP5K] \cdot V_{cytosol})}{dt} = -(k1_{("activation\ PIP5K")} \cdot [PIP5K] \cdot [PA] \cdot [smGa])$$

$$+(k1_{("reset\ PIP5K")} \cdot [PIP5Ka])$$

$$\frac{d([P4BP] \cdot V_{Membrane})}{dt} = -V_{Membrane} \cdot ((k1_{("binding\ PI4P")} \cdot [PI4P] \cdot [P4BP] - k2_{("binding\ PI4P")} \cdot [PI4P.P4BP]))$$

$$\frac{d([PI4P.P4BP] \cdot V_{Membrane})}{dt} = +V_{Membrane} \cdot ((k1_{("binding\ PI4P")} \cdot [PI4P] \cdot [P4BP] - k2_{("binding\ PI4P")} \cdot [PI4P.P4BP]))$$

$$\frac{d([R] \cdot V_{Membrane})}{dt} = -V_{Membrane} \cdot (k1_{("activation\ R")} \cdot [L.GPCR] \cdot [R])$$

$$\frac{d([Ra] \cdot V_{Membrane})}{dt} = +V_{Membrane} \cdot (k1_{("activation\ R")} \cdot [L.GPCR] \cdot [R])$$

$$-V_{Membrane} \cdot (k1_{("activation\ Gq")} \cdot [Ra] \cdot [Gq])$$

$$-V_{Membrane} \cdot (k1_{("activation\ G13")} \cdot [Ra] \cdot [G13])$$

$$\frac{d([Gq] \cdot V_{Membrane})}{dt} = -V_{Membrane} \cdot (k1_{("activation\ Gq")} \cdot [Ra] \cdot [Gq])$$

$$\frac{d([RGq] \cdot V_{Membrane})}{dt} = -V_{Membrane} \cdot (k1_{("activation\ PLC")} \cdot [PLC] \cdot [RGq])$$

$$+V_{Membrane} \cdot (k1_{("activation\ Gq")} \cdot [Ra] \cdot [Gq])$$

$$-V_{Membrane} \cdot (k1_{("inactivation\ Gq")} \cdot [RGq])$$

$$\frac{d([G13] \cdot V_{Membrane})}{dt} = -V_{Membrane} \cdot (k1_{("activation\ G13")} \cdot [Ra] \cdot [G13])$$

$$\frac{d([RG13] \cdot V_{Membrane})}{dt} = +V_{Membrane} \cdot (k1_{("activation\ G13")} \cdot [Ra] \cdot [G13])$$

$$-V_{Membrane} \cdot (k1_{("inactivation\ G13")} \cdot [RG13])$$

We first estimated the overall rate of the PA and Ins conversion into PI ($k_1$, Figure 2B), using the long-term recovery rate of Ins after activation [7] (Supplementary Figures S1, S2), which provided an entry point to the PI cycle. We calculated that the rate of the conversion was around 1 molecule of Ins/CDIPT/second. The CDIPT/Nir2/CDS complex was reduced to the simple "PA + Ins + CDIPT -> PI + CDIPT" reaction. We reasoned that as CDIPT was the least abundant protein in the complex and with no other proteins capable of doing a similar conversion, its activity would govern (i.e. limit) the rate of the entire complex. We then reasoned that, in inactive cells, the rates of PA and Ins production by PLCβ had to be in equilibrium with their consumption by CDIPT ($k_2$, Figure 2B, Supplementary Figure S2A, mostly due to random and limited activation events from GPCR ligands present in the blood circulation). Parameter estimation and parameter scans were performed in COPASI to estimate the reaction rate constants and match the experimental data in inactive cells.

**Model Iteration 2: Activated PI cycle**

The activation of the PLCβ-led cycle is downstream of a series of reactions involving the ligand (L) binding to the transmembrane receptor (R) and activating it, followed by the binding of Gαq proteins at the membrane (Supplementary Figure S2B). The complex (RGq) then activates PLC (PLCa). This activated state is, however terminated rapidly, releasing low activity PLCβ and an inactive GPCR complex (RGi). PLCβ activity, via calcium release and PKC activation, eventually leads to the activation of a number of downstream proteins such as small G proteins (smG) and cPLA2. As a simplification we used PLCa as the trigger for the activation of both of these targets.

**Coincidence detection during platelet activation**

The change of activity of the enzymes regulating the PI cycle is essential in our model to switch from unstimulated to stimulated states, and back again. Although this is dependent on

receptor activation, literature suggests that coincidence detection, where several regulatory molecules act in concert, is a key mechanism in fine tuning the signalling responses. PIP5K activation for instance is achieved via PA and small G proteins [39]. In our model, PA alone could not control the regulation of these enzymes as it is present at low levels before activation and would trigger enzyme activation inappropriately. Instead, activated smG (smGa) together with PA creates a coincidence detection circuit leading to the activation of the kinase only after receptor stimulation ($k_3'$, Fig 2B, Supplementary Figure 2A, C). We also tested that activation would lead to different results when being triggered directly by Gαq-coupled receptors or via PLC proteins, but our model led to virtually identical simulations (Supplementary Figure S3).

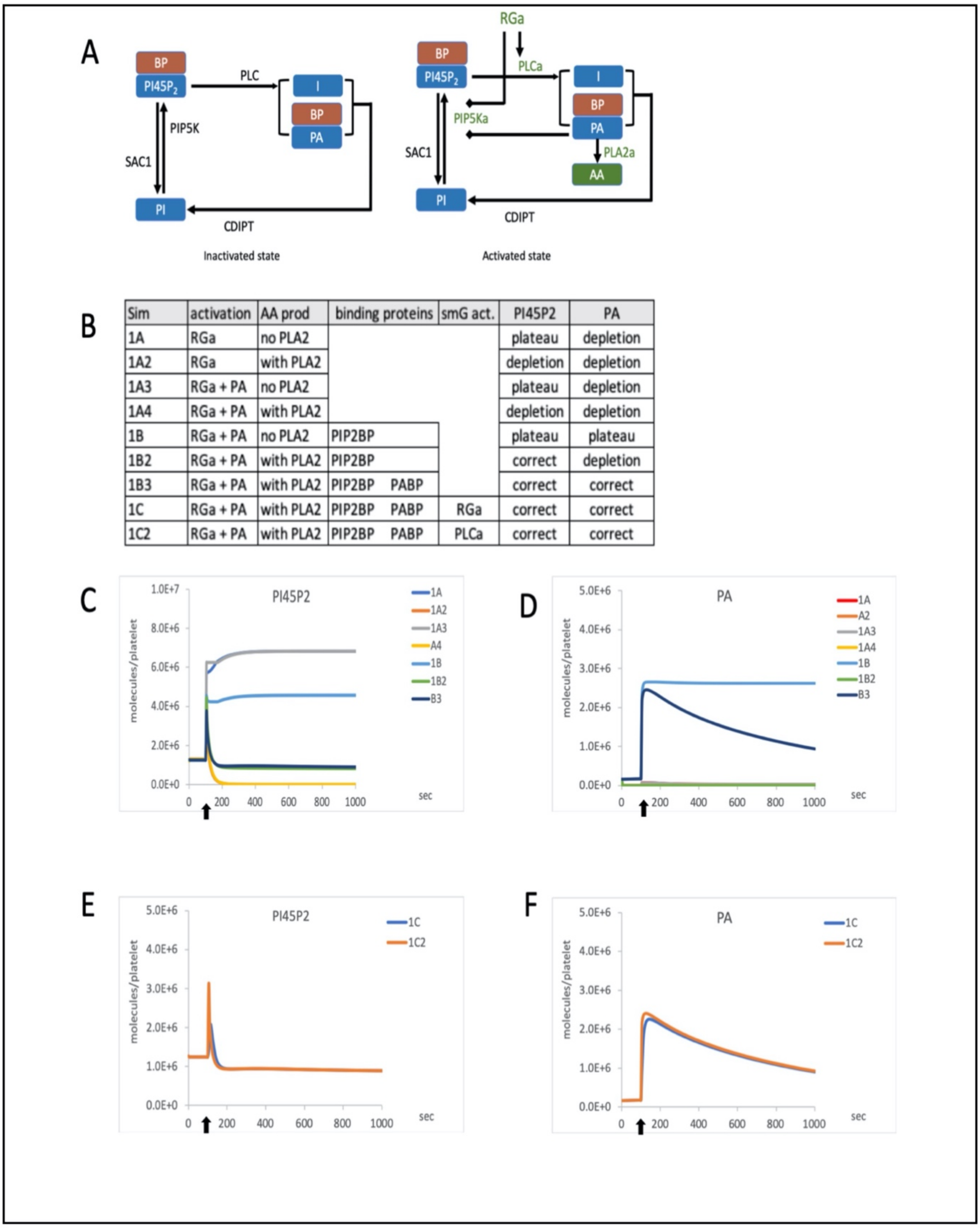


| Sim | activation | AA prod | binding proteins | smG act. | PI45P2 | PA |
|---|---|---|---|---|---|---|
| 1A | RGa | no PLA2 | | | plateau | depletion |
| 1A2 | RGa | with PLA2 | | | depletion | depletion |
| 1A3 | RGa + PA | no PLA2 | | | plateau | depletion |
| 1A4 | RGa + PA | with PLA2 | | | depletion | depletion |
| 1B | RGa + PA | no PLA2 | PIP2BP | | plateau | plateau |
| 1B2 | RGa + PA | with PLA2 | PIP2BP | | correct | depletion |
| 1B3 | RGa + PA | with PLA2 | PIP2BP PABP | | correct | correct |
| 1C | RGa + PA | with PLA2 | PIP2BP PABP | RGa | correct | correct |
| 1C2 | RGa + PA | with PLA2 | PIP2BP PABP | PLCa | correct | correct |

**Supplementary Figure S3. Description of the first iterations of the core model.**
a: Graphical summary of the reactions in the early iterations for either inactivated or activated states. Activated GPCRs (RGa) trigger the activation of the PIP5-kinase (PIP5Ka) together with PA. The levels of PA in the cells are also regulated by activated cPLA2 (PLA2a) which produces arachidonic acid (AA), a precursor of Prostaglandin H2 (itself a precursor of TxA2 produced by the platelet as a secondary signaling molecule) and several eicosanoids. b: Table summarising the different steps in the early core model construction and the output for

PI45P2 and PA. c-d: Graphs of the results shown in the table for the simulations 1A-B. e-f: Graphs of the results shown in the table for each simulation 1C. the results were virtually identical whether the activation of PIP5K was simulated directly via the activated GPCR or indirectly by the activated PLC (PLCa). Activation time points are indicated by arrows.

Ins and PA, while being used in equal amounts for PI regeneration, show different dynamics after activation, with PA being removed more quickly than Ins. This suggests that an additional reaction is being activated. We thus added a set of reactions that results in the activation of the enzyme cPLA2 and which modifies PA to produce arachidonic acid (AA), a precursor of Prostaglandin H2, itself a precursor of TxA2 and several eicosanoids [40]. Our model does not include a specific termination step for cPLA2 as the experimental results in platelets show that PA levels are still falling 30 minutes after activation[8] (1800 seconds, Supplementary Figure S1)

Experimental data show that the levels of PI and PI45P2 (or PI4P) are in a 3:1 equilibrium within the plasma membrane. This suggests that the PI to PI45P2 reaction is 3 times slower than the reverse reaction in an inactive cell. After activation, the plasma membrane is rapidly depleted of PI due to the accelerated rate $k_3$'. In contrast, $k_4$ is not drastically changed as there is no evidence of PI being regenerated from PI45P2/PI4P more rapidly after activation, than before.

Although PI45P2 and PA pre-activation steady-states could be simulated in most cases as well as the peaks of production following the activation event, the new equilibrium observed experimentally after the activation peak could not, however, be correctly simulated in iteration 2, and both PLs were severely depleted (Supplementary Figure S3C-D). Adapting the rates of production or recycling of the different PL did not lead to any biologically valid solution suggesting the problem was actually linked to the availability of PL to the enzymes, rather than the rates at which they were being used in the cycle.

**Model Iteration 3: Phospholipid binding proteins**

The addition of PL binding proteins (PLB for PI45P2, PABP for PA), which restrict the availability of the PL to the different enzymes of the PI cycle, was tested (Supplementary Figure S3C-D) in this model iteration. The PI45P2-binding protein numbers and on/off binding rates $k_6$ and $k_7$ were then estimated using parameter scans. Once these steps were added we were able to stabilise the levels of PI45P2 after activation and simulate the experimentally observed equilibrium after activation (Figure 3B, Supplementary Figure S3). Furthermore, the PI45P2-binding protein numbers predicted by our model matched with the values obtained from mining the proteome dataset using UniProt data at around 1-2 x$10^6$ molecules per cell (Supplementary Table S6).

We also observed that the number of PI45P2-binding proteins corresponds to the values of PI45P2 before and after activation. As the identity of PA, PI4P and other DAG binding proteins has not been yet fully characterised, we used the latter observation to estimate their amounts for our model. We also kept the PI45P2-binding proteins on/off rates computed values for each new Pl-binding reaction ($k_8$-$k_{12}$).

**Table S6:**

PIP3 BINDING PROTEINS: 69374

| Gene Name | Uniprot | Hs plt copy nb |
|---|---|---|
| Acap1 | Q15027 | 1200 |
| Acap2 | Q15057 | 1100 |
| Akt1 | P31749 | 2000 |
| Akt2 | P31751 | 2200 |
| Appl2 | Q8NEU8 | 5000 |
| Arap1 | Q96P48 | 3100 |
| Arhgef7 | Q14155 | 3100 |
| Btk | Q06187 | 11100 |
| Cyth1 | Q15438 | 1080 |
| Cyth2 | Q99418 | 1600 |
| Cyth3 | O43739 | 500 |
| Dapp1 | Q9UN19 | 4200 |
| Elmo1 | Q92556 | 1600 |
| Elmo2 | Q96JJ3 | 500 |
| Esyt2 | A0FGR8 | 3500 |
| Gab1 | Q13480 | 1252 |
| Gab2 | Q9UQC2 | 371 |
| Gab3 | Q8WWW8 | 371 |
| Mapkap1 | Q9BPZ7 | 1200 |
| Myo1c | O00159 | 2700 |
| MYO1E | Q12965 | 590 |
| MYO1F | O00160 | 640 |
| Myo1g | B0I1T2 | 1000 |
| Pdpk1 | O15530 | 1000 |
| Phldb1 | Q86UU1 | 100 |
| Rasa3 | Q14644 | 8300 |
| Skap2 | O75563 | 6800 |
| Snx9 | Q9Y5X1 | 1900 |
| Tbc1d2b | Q9UPU7 | 70 |
| Tec | P42680 | 1300 |

PI(4,5)P2 BINDING PROTEINS: 1118820

| Gene Name | Uniprot | Hs plt copy nb |
|---|---|---|
| ACTN1 | P12814 | 92100 |
| ACTN2 | P35609 | 25800 |
| ACTN4 | O43707 | 45600 |
| ADDA | P35611 | 3900 |
| ANXA1 | P04083 | 810 |
| ANXA4 | P09525 | 2900 |
| ANXA7 | P20073 | 4900 |
| ARF4 | P18085 | 33300 |
| ASAP1 | Q9ULH1 | 3600 |
| ASAP2 | O43150 | 4100 |
| DEST | P60981 | 14400 |
| EX3L2 | Q2M3D2 | 1800 |
| EXOC1 | Q9NV70 | 1000 |
| EXOC2 | Q96KP1 | 1800 |
| EXOC3 | O60645 | 1600 |
| EXOC4 | Q96A65 | 1900 |
| EXOC6 | Q8TAG9 | 710 |
| EZRI | P15311 | 13300 |
| FIG4 | Q92562 | 630 |
| GELS | P06396 | 52900 |
| LIMS1 | P48059 | 49800 |
| MARK2 | Q7KZI7 | 1100 |
| MOES | P26038 | 34800 |
| PIPNB | P48739 | 2500 |
| PROF1 | P07737 | 503000 |
| SDCB1 | O00560 | 770 |
| STX7 | O15400 | 7100 |
| TAGL2 | P37802 | 130000 |
| VILI | P09327 | 1600 |
| VINC | P18206 | 81100 |

PA BINDING PROTEINS 391440

| Gene Name | Uniprot | Hs plt copy nb |
|---|---|---|
| ACAP1 | Q15027 | 1200 |
| ACAP2 | Q15057 | 1000 |
| AGAP2 | Q99490 | 1200 |
| ARF1 | P84077 | 49800 |
| ARF6 | P62330 | 6400 |
| ASAH1 | Q13510 | 2600 |
| ASAP1 | Q9ULH1 | 3600 |
| CTBP1 | Q13363 | 800 |
| DPYSL2 | Q16555 | 5400 |
| DYN1 | Q05193 | 5200 |
| EHD3 | Q9NZN3 | 18600 |
| FER | P16591 | 1100 |
| FGR | P09769 | 2600 |
| GDI1 | P31150 | 21700 |
| GSDMD | P57764 | 1500 |
| IQGAP1 | P46940 | 1000 |
| KIF5B | P33176 | 3000 |
| KS6C1 | Q96L93 | 590 |
| MBP | P02686 | 850 |
| MILK1 | Q8N3F8 | 670 |
| MTMR6 | Q9Y217 | 1200 |
| MTOR | P42345 | 1000 |
| NF2 | P35240 | 930 |
| NRGN | Q92686 | 11800 |
| NSF | P46459 | 3500 |
| OPA1 | O60313 | 2100 |
| OSBL1 | Q9BXW6 | 600 |
| OSBL2 | Q9HIP3 | 1100 |
| PACSIN2 | Q9UNF0 | 3900 |
| PARD3 | Q8TEW0 | 1300 |
| PI5K1C | O60331 | 1200 |
| PITC1 | Q9UKF7 | 1600 |
| PLTP | P55058 | 620 |
| PPP1CC | P36873 | 13200 |
| PRKCE | Q02156 | 670 |
| RAB3A | P20336 | 3700 |
| RAPGEF2 | Q9Y4G8 | 870 |
| RAPGEF6 | Q8TEU7 | 590 |
| RPS6KB1 | P23443 | 500 |
| SDCBP | O00560 | 770 |
| SESTD1 | Q86VW0 | 1300 |
| SH3K1 | Q96B97 | 1100 |
| PTPN6 | P29350 | 8900 |
| SIN1 | Q9BPZ7 | 1200 |
| SOS1 | Q07889 | 780 |
| SPHK1 | Q9NYA1 | 1200 |
| SRC | P12931 | 20000 |
| YWHAZ | P63104 | 177000 |
| ATP13A2 | Q9NQ11 | TiO2 |
| COMD1 | Q8N668 | TiO2 |
| DOCK1 | Q14185 | TiO2 |
| MARCKL | P49006 | TiO2 |

OTHER

| Gene Name | Uniprot | Hs plt copy nb |
|---|---|---|
| ESYT1 | Q9BSJ8 | 4600 |
| ESYT2 | A0FGR8 | 3500 |
| SYTL4 | Q96C24 | 9200 |
| ARRB1 | P49407 | 6200 |
| ARRB2 | P32121 | 1000 |
| SNX1 | Q13596 | 1500 |
| SNX12 | Q9UMY4 | 5100 |
| SNX15 | Q9NRS6 | 1000 |
| SNX16 | P57768 | 670 |
| SNX17 | Q15036 | 1400 |
| SNX2 | O60749 | 3600 |
| SNX24 | Q9Y343 | 1800 |
| SNX27 | Q96L92 | 660 |
| SNX29 | Q8TEQ0 | 810 |
| SNX3 | O60493 | 8500 |
| SNX30 | Q5VWJ9 | 1100 |
| SNX4 | O95219 | 1400 |
| SNX5 | Q9Y5X3 | 1700 |
| SNX6 | Q9UNH7 | 2300 |
| SNX8 | Q9Y5X2 | 730 |
| SNX9 | Q9Y5X1 | 1900 |

**Supplementary Table S6:** Quantification of Lipid binding Proteins in human platelets. The protein numbers are expressed as copies per cell.

## Model iteration 4: Extending the Core Model

One aim of our model is for it to be the starting point for more in-depth computational analysis of the different molecular steps in a signalling cascade. Our model design allows for expansion of some segments within the range of input and output constraints defined in the current iteration. To test whether we could easily expand some parts of the model we introduced several key PL (namely PI4P and DAG) and IP3 and their associated enzymes.

The addition of PI4P, together with the binding proteins P4B, and the OCRL1 and PI4K enzymes suggested that after activation the rates of these two enzymes ($k_{14}$', $k_{16}$' respectively) had to be increased in a manner similar to PIP5K ($k_{15}$') to maintain the equilibrium between PI4P and PI45P2. Furthermore, the addition of the PI4P step led to a higher level of PIP2 after activation, leading to only about 10% loss of PIP2, well within the range of the experimental data (Figure 3). In contrast, the inclusion of SAC1 as a recycling reaction of PI from PI4P remains the same before and after activation ($k_{17}$).

Upon thrombin stimulation, the PLCβ-dependent pathway is immediately activated as indicated by the peaks of IP3 and DAG within 5 seconds and is completed within 30 seconds with the plateauing of both Ins and PA. DAG is converted into PA by diacylglycerol kinase (DGK) enzymes, with a reverse reaction driven by Phosphatidic Acid Phosphatases (PAP) enzymes ($k_{18}$, $k_{19}$). In the case of human platelets, only one type of PAP was found in the proteomic dataset, Lipin2 (labelled LPP in our model), at a very low level, suggesting that this reaction may be limited in these cells. IP3 is removed very quickly from the cytoplasm before being recycled into Ins ($k_{20}$, $k_{21}$).

Lastly, experimental evidence suggest that the PI cycle and PLCβ intermediates find a new equilibrium or return to their initial concentration within 2 minutes of the activation event, and our model has thus been designed to inactivating the GPCR complex ($k_{30}$, see Supplementary Table S3) and rapidly returning PLCβ, PI4K, PIP5K, OCRL1 and PLA2 to their pre-activated activity rates ($k_{31}$ *to* $k_{35}$, see Supplementary Table S3) as demonstrated in other mammalian cells [22,23].

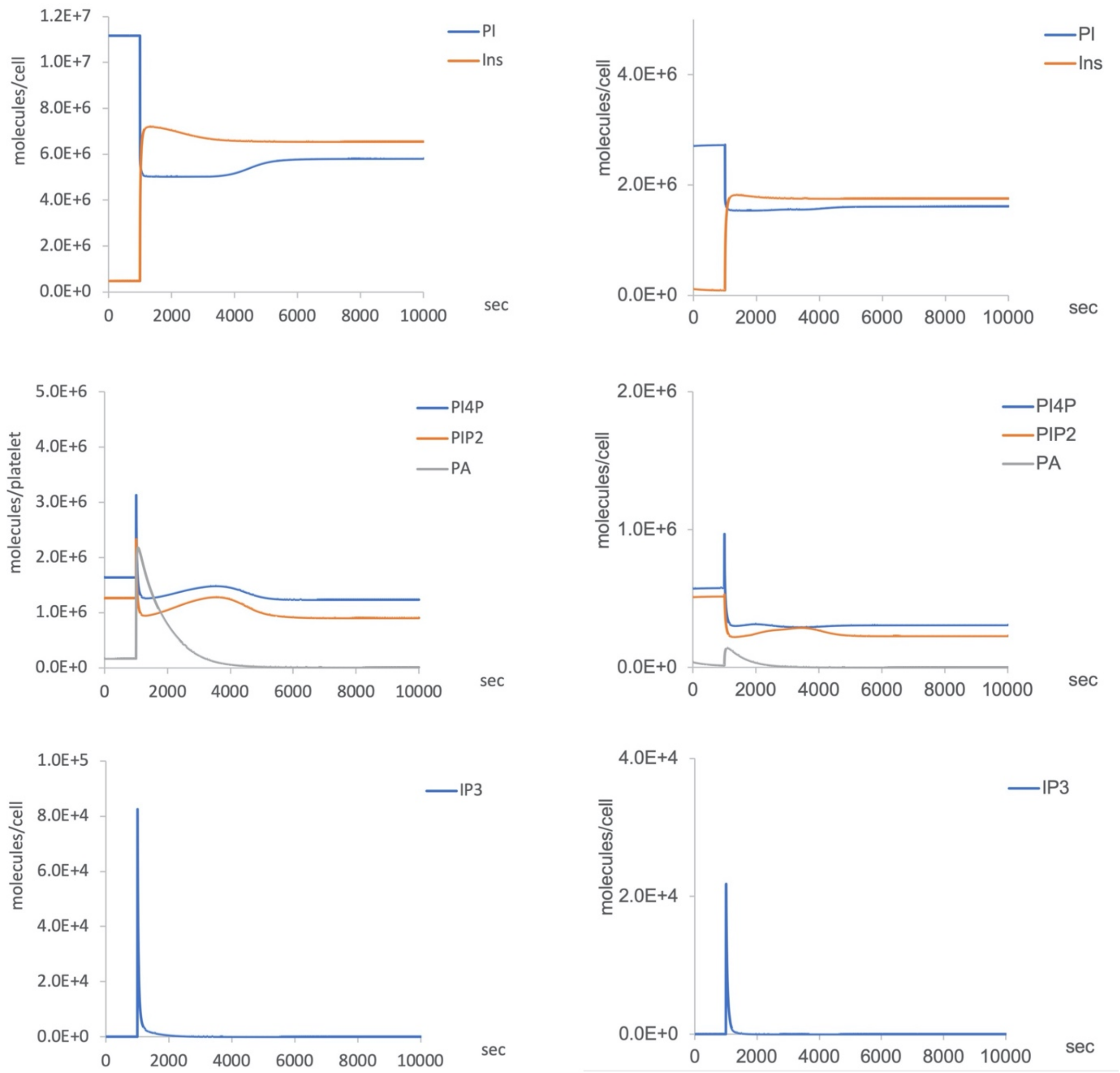


**Supplementary Figure S4. Comparison of the core model outputs in human and mouse platelets.** The volume of the mouse platelet, PL and IP initial concentrations have been modified as described in the material and methods. The numbers of GPCR receptors for each platelet type are listed in Supplementary Table S4. The overall results for the PL, Ins and IP3 are virtually identical except for the overall levels which are related to the initial amounts in the two cell types. The simulations have been extended to 10000 seconds to capture any late trend, with the activation occurring at 1000 sec (arrows).

**Table S7:**

**A:**

| Protein | Platelet Hs | Platelet Mm | HeLa | U2OS* | Pltx17** |
|---|---|---|---|---|---|
| CDIPT | 1100 | 5358 | 160000 | 2289 | 18700 |
| DGK | 6600 | 3215 | 8440 | 4283 | 112200 |
| Ga13 | 6100 | 8506 | 41000 | 17235 | 103700 |
| Gaq | 14800 | 18583 | 22000 | 20000 | 251600 |
| IP3E | 3000 | 2923 | 11407 | 2000 | 51000 |
| LPP | 100 | 590 | 50000 | 2178 | 1700 |
| OCRL1 | 850 | 1039 | 40500 | 15000 | 14450 |
| PI4K | 1800 | 1776 | 2600 | 2500 | 30600 |
| PIP5K | 1200 | 1190 | 15000 | 5335 | 20400 |
| cPLA2 | 3100 | 8047 | 120000 | 10000 | 52700 |
| PLCb | 5000 | 1793 | 22630 | 4913 | 85000 |
| Rac | 32900 | 5261 | 459000 | 474847 | 559300 |
| Sac1 | 10500 | 5866 | 152467 | 10638 | 178500 |

* numbers in red were estimated using parameter scans
** calculated values from human platelet data

| Receptor | Platelet Hs | Platelet Mm |
|---|---|---|
| Tpa | 1500 | 1942 |
| PAR1 | 1276 | |
| PAR3 | | 3825 |
| PAR4 | 1100 | 5195 |
| P2Y1 | 150 | 983 |
| P2Y12 | 400 | 2681 |

**B:**

| | **human platelet** | **large cell sim** | **mouse platelet** |
|---|---|---|---|
| **total volume** | 7 fl | 2000 fl | 3.5 fl |
| **plasma membrane** | 1 fl | 17 fl | 0.25 fl |
| **reaction cytosol** | 1 fl | 17 fl | 0.25 fl |
| **Gq-coupled Receptors** | 5000 | 85000 | 11945 |

**Supplementary Table S7:** A: Protein numbers in HeLa, U2OS, mouse platelets, compared to human platelets. All data from respective proteome datasets unless stated otherwise. The protein numbers are expressed as copies per cell. B: reaction volumes and Gαq-coupled receptor numbers for each cell type simulations.

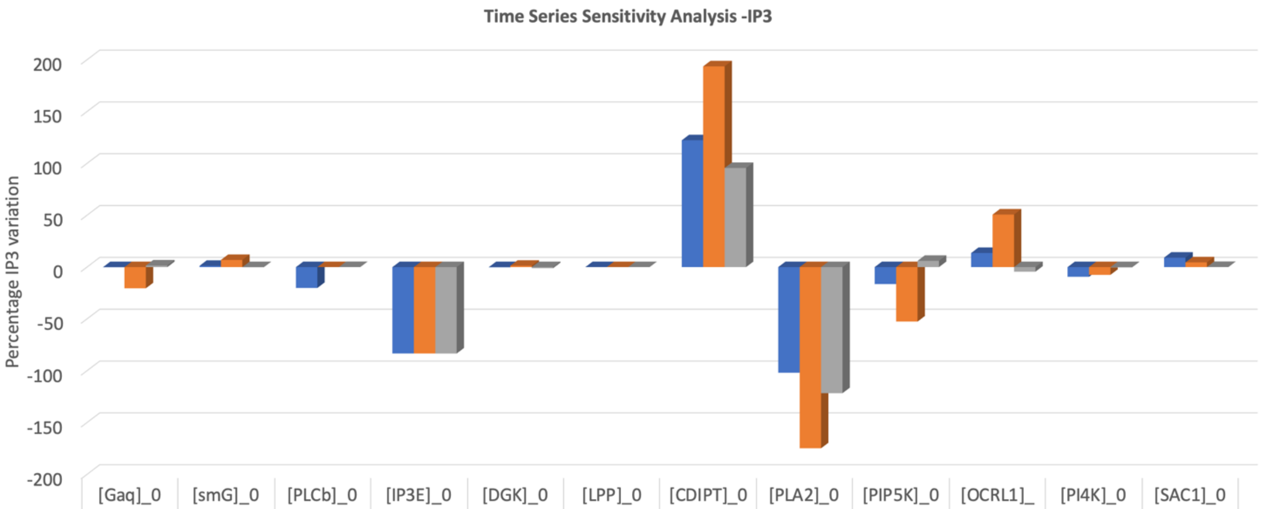


| | [Gaq]_0 | [smG]_0 | [PLCb]_0 | [IP3E]_0 | [DGK]_0 | [LPP]_0 | [CDIPT]_0 | [PLA2]_0 | [PIP5K]_0 | [OCRL1]_0 | [PI4K]_0 | [SAC1]_0 |
|---|---|---|---|---|---|---|---|---|---|---|---|---|
| U2OS | -0.13 | 1.22 | -20.09 | -83.31 | -0.17 | 0.00 | 122.36 | -101.90 | -16.39 | 13.62 | -9.35 | 9.12 |
| HeLa | -20.41 | 6.91 | -0.01 | -83.28 | 1.32 | 0.00 | 193.67 | -174.78 | -52.53 | 50.72 | -7.47 | 4.57 |
| Plt17 | 1.54 | -0.15 | 0.03 | -83.33 | -1.01 | 0.00 | 95.62 | -121.63 | 6.04 | -4.39 | -0.34 | 0.09 |

**Supplementary Figure S5. Comparison of Sensitivity Analyses for IP3 in nucleated cells simulations.** Time series Sensitivity Analyses of the impact of some key protein initial concentrations on IP3 outputs were performed and compared when our model was populated with either human platelet, HeLa or U2OS proteomic data. We used a delta factor of 0.2 that covers the range of proteomic estimate errors observed with the results of the human platelet proteome. We used protein numbers estimated via Parameter Scans for missing protein values in the U2OS proteomic dataset namely G$\alpha$q, cPLA2, PI4K, OCRL1 and IP3 modifying enzymes (IP3E). IP3 results for each protein initial concentration are shown in the table.
